# Surgical management of Helveston syndrome (Triad exotropia)

**DOI:** 10.1007/s10792-021-02027-1

**Published:** 2021-11-08

**Authors:** Xiaoqin Jin, Yi Peng, Samer Abdo Al-wesabi, Jun Deng, Yue Ming, Xi Wu

**Affiliations:** 1 Department of Strabismus and Pediatric Ophthalmology, Wuhan Aige (Eyegood) Eye Hospitals, No. 403 Fazhan Avenue, Jiangan District, Wuhan, 430019 China; 2 Department of Ophthalmology, Peking University People’s Hospital, No. 11 Xizhimen South Street, Xicheng District, Beijing, 100044 China

**Keywords:** Helveston syndrome, Triad exotropia, Surgical planning, Dissociated vertical deviation (DVD), A-pattern, Superior oblique muscle overaction (SOOA), Superior oblique muscle lengthening

## Abstract

**Purpose:**

To evaluate and compare different surgical approaches for the treatment of Helveston syndrome and provide further information for preoperative planning.

**Methods:**

From February 2008 to December 2018, data of 52 patients with Helveston syndrome were retrospectively reviewed. Different surgical approaches were selected based on the extent of A-pattern exotropia, dissociated vertical deviation (DVD), and both superior oblique muscle overaction (SOOA) with fundus photograph intorsion. Eye position, A-pattern, DVD, superior oblique muscle function, and binocular vision function were evaluated pre- and postoperatively. The average follow-up duration was 20.5 months.

**Results:**

Nine cases underwent simultaneous horizontal deviation correction with bilateral superior rectus recession, 24 underwent simultaneous horizontal deviation correction with bilateral superior oblique muscle lengthening, and 19 underwent two stages of horizontal deviation correction with superior oblique muscle lengthening, and later bilateral superior rectus recession. A-pattern, DVD, SOOA, and fundus intorsion were all collapsed in all patients postoperatively. Forty-five patients had an orthophoric eye position with considerably aligned ocular movements postoperatively. The total success rate was 86.5%. Postoperatively, eight of the 10 patients with diplopia experienced a recovery of binocular single vision and three had a recovery of rudimentary stereopsis (Titmus 3000–400 s of arc). The compensatory head posture of patients improved significantly postoperatively.

**Conclusions:**

The surgical planning of Helveston syndrome should be designed based on the degree of the A-pattern, SOOA, DVD, and the intorsion in fundus photographs, and the appropriate approach should be selected to improve patient satisfaction.

## Introduction

Helveston syndrome or triad exotropia was first described by Helveston in 1969; it is characterized by a triad of A-pattern exotropia, dissociated vertical deviation (DVD), and superior oblique overaction (SOOA) [1].

The decision of surgical management of this triad is quite challenging; the treatment plan aimed at achieving postoperative ocular movement coordination should consider the possible complications and unpredictable outcomes of the surgery. However, existing reports suggest different surgical approaches and variable outcomes of each surgery [2–7].

In the current study, different surgical approaches were used in managing 52 cases of Helveston syndrome that were reviewed retrospectively. We report our experience of exploring the surgical effects and provide further information for preoperative planning.

## Materials and Methods

### Subjects

The study was conducted in accordance with the Declaration of Helsinki. Patients or their parents signed informed consent forms before the surgery. All patients underwent strabismus correction for the first time from February 2008 to December 2018. Patients with a history of previous strabismus surgery, presence of any cranial nerve palsy, restricted eye movement or a postoperative follow-up less than 12 months were excluded.

The patients were divided into three groups based on the degree of the A-pattern, SOOA, DVD, and the intorsion in fundus photographs. Different surgical methods were used in each group as shown in Table 1. The surgical planning for patients in group A was mainly aimed at solving the DVD by superior rectus recession, while that in groups B and C was mainly to resolve the DVD and SOOA by superior oblique lengthening. In group A (9 cases), patients underwent simultaneous horizontal deviation correction with bilateral superior rectus recession; group B (24 cases), horizontal deviation correction with bilateral superior oblique muscle lengthening; and group C (19 cases), initial horizontal deviation correction with superior oblique muscle lengthening, followed by a bilateral superior rectus recession after 3 months or more, to correct the DVD.

**Table 1:** Classification of patients according to the A-pattern, DVD, SOOA, and fundus intorsion

Groups (n)	A-pattern	DVD	Fundus photography intorsion	SOOA
Group A: horizontal deviation correction and bilateral superior rectus recession (9)	≈ 10Δ	> 15Δ	No/yes	0 to + 1
Group B: horizontal deviation correction with superior oblique muscle lengthening (24)	> 15Δ	≤ 10Δ	Yes	≥ + 2
Group C: horizontal deviation correction with superior oblique muscle lengthening + bilateral superior rectus recession 3 months later (19)	10Δ–15Δ	> 15Δ	Yes	≥ + 2

*Δ* prism diopter, *DVD* dissociated vertical deviation, *SOOA* superior oblique overaction

### Inspection methods

All patients underwent complete ophthalmic evaluation to exclude any other ocular abnormalities, and received an orthoptic evaluation by a strabismus specialist (blinded to the data that were going to be used in the study) preoperatively; at day 1, week 1, and 1 month postoperatively; and on subsequent visits as required. An alternative prism cover test was used for left and right eye fixation. The measurements were obtained after correction of the refractive errors in the primary position and lateral gazes, and in up and down gaze of 15°, as well as with head tilted right and left, at near and at distance. SOOA was measured according to the 9-point system (− 4 to + 4) [8]. All patients were examined by a synoptophore at nine diagnostic positions of gaze, and by the Titmus test for stereo-acuity. Eye intorsion was evaluated based on the angle between the two lines of the fovea and the lower one-third of the optic disc in fundus photography [9]. The eye was termed intorted if the angle was greater than 9 degrees above the bottom of the disc.

A successful surgical outcome was defined as a reduction in the A-pattern to ≤ 8PD, a vertical deviation of ≤ 5PD in the primary position coupled with the absence of symptoms attributable to residual deviation, and a reduction in SOOA and fundus intorsion.

### Statistical analysis

A paired t test was used for the statistical analysis between the average pre- and postoperative results.

#### Operative methods

All surgeries were performed using a microscope by a single surgeon (XQJ). The objective forced duction test was performed in all patients perioperatively to determine the tension (pulling force) of the superior oblique (SO) muscle and compare it with that of the contralateral eye.

Lateral rectus recession was chosen for the correction of horizontal deviation using the conventional method with 6/0 Vicryl absorbable sutures based on the degree of lateral deviation. The lateral and medial recti were recessed and resected for the non-fixing amblyopic eyes when no superior rectus recession was made on the same eye. The superior rectus was recessed based on the degree of DVD in each eye; the muscle was recessed 7 mm when DVD is ≤ 15PD, 8 mm when DVD 15PD–25PD, and 8 mm with posterior fixation at 6 mm behind the re-attachment position when DVD > 25PD. The suture spacer procedure was used for SO lengthening. SOOA and positive objective forced duction test, as well as fundus intorsion, were considered for SO lengthening. When SOOA was + 1 and fundus intorsion is ≤ 10°, the tendon was lengthened to 4 mm; SOOA + 2 and fundus intorsion of 11–15°, lengthened to 5 mm; SOOA + 3 and fundus intorsion of 16–20°, lengthened to 6 mm; and SOOA + 4 with fundus intorsion of > 20°, lengthened to 7–8 mm.

The tendon of the SO was exposed in the upper temporal quadrant, at the lateral border of the superior rectus. Using two muscle hooks stretching the tendon of the SO, two double-armed 5–0 non-absorbable sutures were inserted and locked to the anterior and posterior parts of the tendon insertion and the slipknot was sutured. Forced duction was performed again, and the amount of lengthening was adjusted to ensure the balance between the tension of the SO and inferior oblique on the same eye. Both eyes were pulled again to feel the tension of the SO on both sides; then, the slipknot was altered into a double square knot. The length of the desirable extension away from the knot was then measured. Straight surgical pliers were used to fix the measured endpoint and to tie a knot above the pliers. The amount of lengthening was between the two knots. The tendon was cut between the two set sutures, and a forced test was performed to ensure that the tension of the SO was relieved; next, the remaining sutures were cut.

## Results

Fifty-two patients with the triad of Helveston syndrome underwent surgical correction (32 males (64 eyes) and 20 females (40 eyes), aged between 4 and 25 years). Amblyopia was detected in six cases (7 eyes) and latent nystagmus in 10 cases (20 eyes). Ten cases had a complaint of diplopia and eight had a compensatory head posture. The follow-up duration ranged from 12 to 48 months, with an average of 20.5 months. The postoperative measurements were taken in the last follow-up visit. A-pattern, DVD, SOOA, and fundus intorsion were all collapsed in all patients postoperatively (Table 2).

**Table 2:** Pre- and postoperative measurements of the horizontal deviation, A-pattern, DVD, SOOA, and fundus intorsion (52 patients)

Parameters	Pre-operation M ± SD (Range)	Post-operation M ± SD (Range)	*p* value
Horizontal deviation (PD)	− 40.37 ± 12.16 (− 15 to − 50)	0.45 ± 4.19 (− 10 to 8)	< 0.05*
A pattern (PD)	21.07 ± 12.18 (8–45)	5.58 ± 3.30 (0–11)	< 0.05*
DVD (PD) OD	14.20 ± 6.91 (6–30)	2.47 ± 2.30 (0–8)	< 0.05*
OS	12.13 ± 6.83 (0–35)	2.47 ± 2.12 (0–7)	< 0.05*
SOOA ( +) OD	2.03 ± 0.91 (0–4)	0.11 ± 0.31 (0–1)	< 0.05*
OS	1.33 ± 0.97 (0–4)	0.03 ± 0.19 (0–1)	< 0.05*
Fundus intorsion (°) OD	10.69 ± 5.78 (0–21)	2.43 ± 1.46 (0–5)	< 0.05*
OS	9.62 ± 6.23 (0–20)	2.57 ± 1.65 (0–6)	< 0.05*

DVD: dissociated vertical deviation, SOOA: superior oblique overaction, OD: Right eye, OS: Left eye, PD: prism diopter

**Fig. 1:**
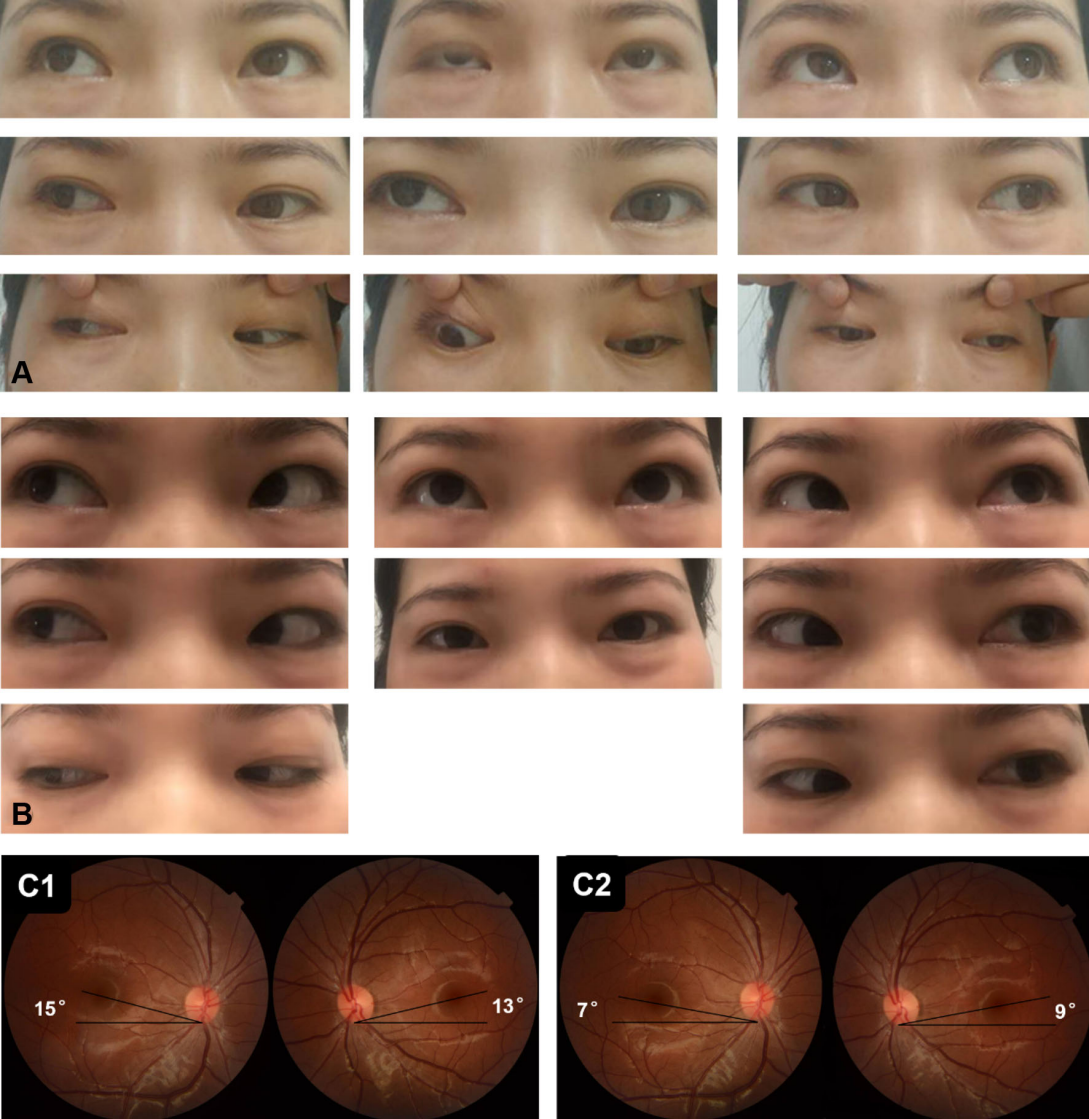
A patient of group (A). **a** Preoperative eye position images; the patient had an A pattern of 10PD, a DVD of 15PD, and fundus intorsion, with no severe SOOA. The patient had horizontal deviation correction and 7 mm bilateral superior rectus recession. **b** Postoperative eye position indicating correction of DVD and A pattern. **c** Fundus photography, C1 demonstrating preoperative intorsion, C2 demonstrating postoperative improvement in intorsion

**Fig. 2:**
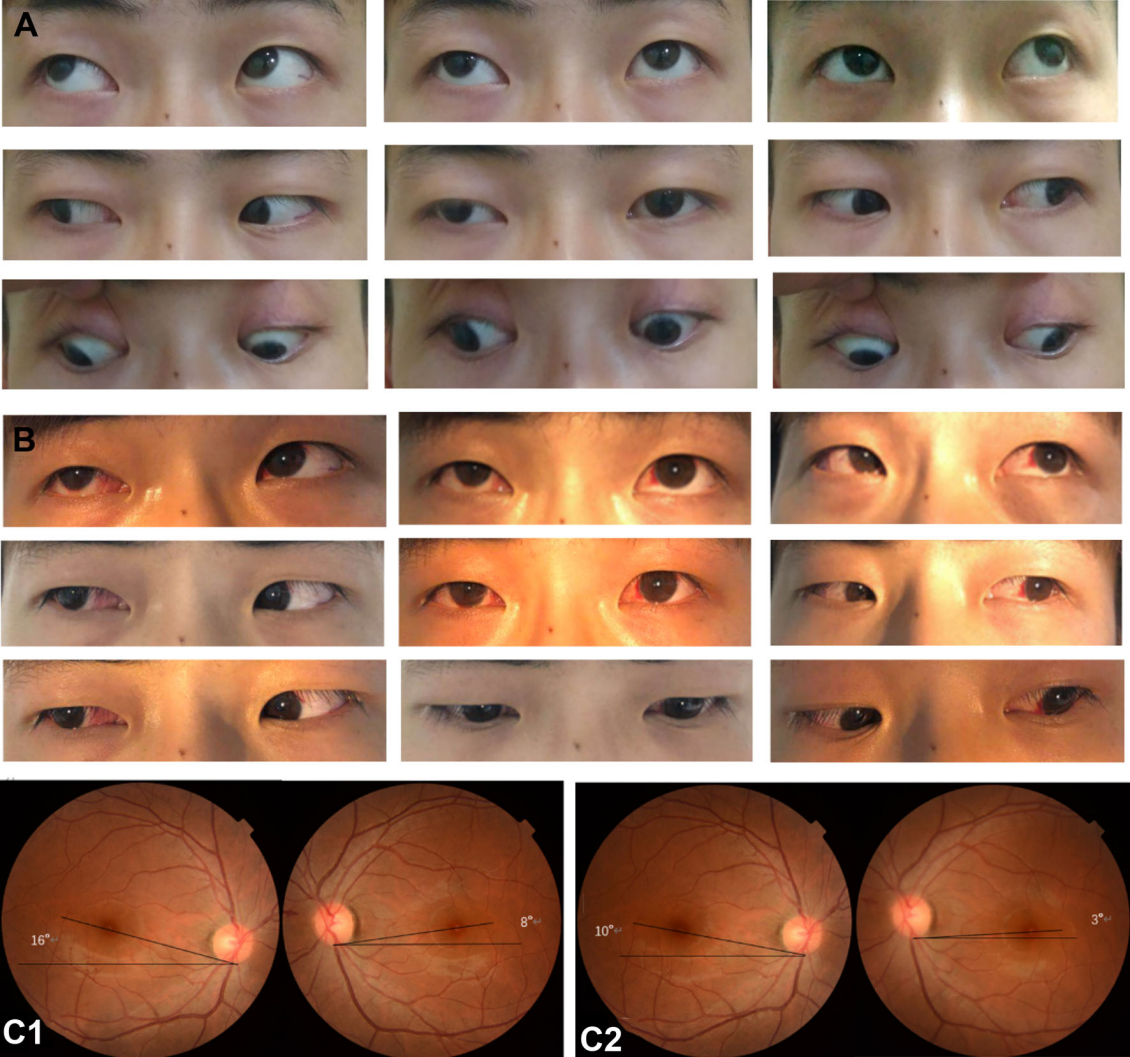
A patient of group (B). **a** Preoperative eye position images; the patient had an A pattern of 30PD, a DVD of 8PD, and a + 2 SOOA with fundus intorsion. The patient had horizontal deviation correction with superior oblique muscle lengthening. **b** Postoperative imaging showing correction of the DVD and A pattern. **c** Fundus photography, C1 demonstrating preoperative intorsion, C2 demonstrating postoperative improvement in intorsion

**Fig. 3:**
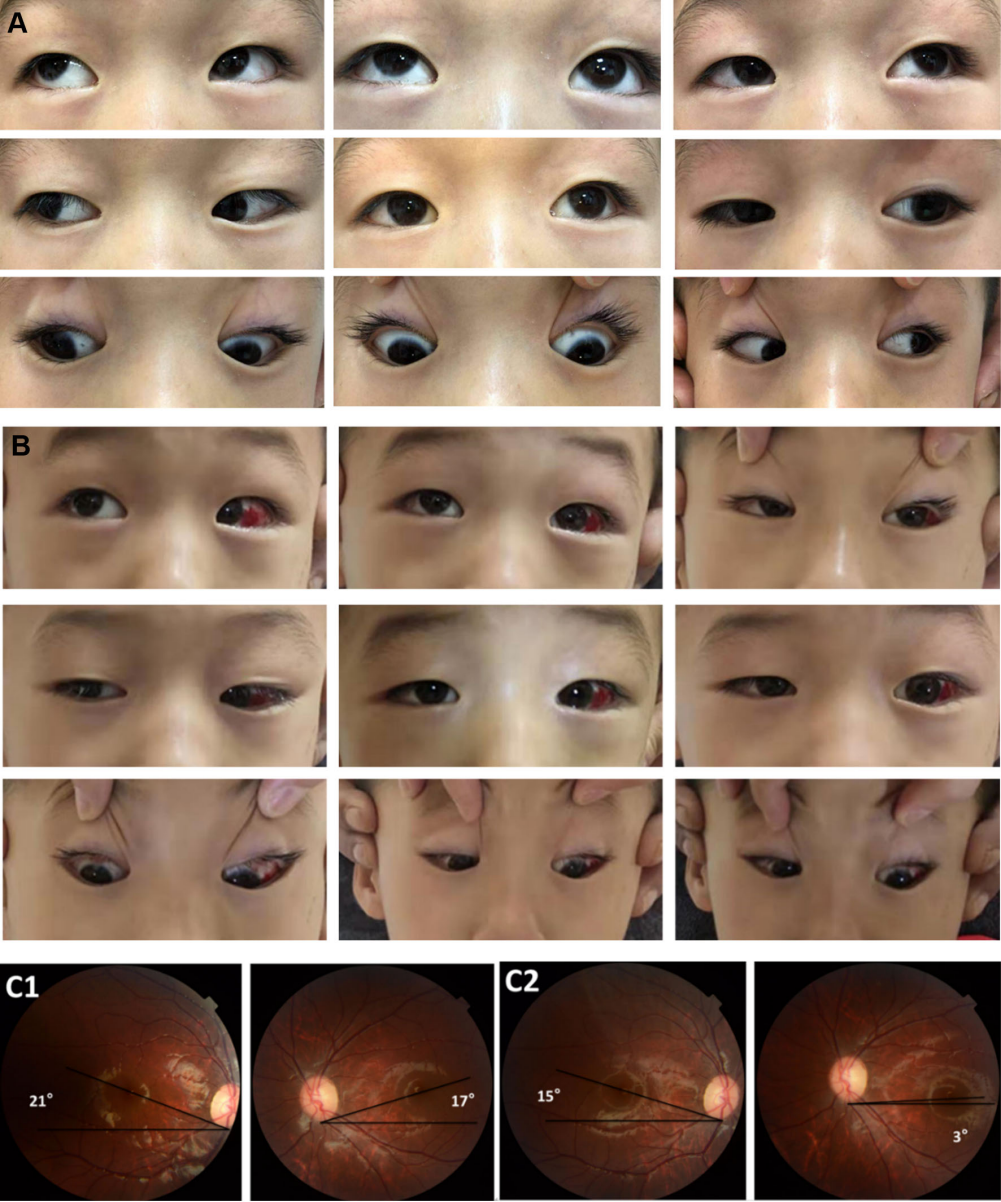
A patient of group (C). **a** Preoperative eye position images; the patient had an A pattern of 10PD, a DVD of 20PD, and a + 3 SOOA with significant fundus intorsion. The patient had horizontal deviation correction with superior oblique muscle lengthening and superior rectus recession. **b** Postoperative imaging showing correction of the DVD and A pattern. **c** Fundus photography, C1 demonstrating preoperative intorsion, C2 demonstrating postoperative improvement in intorsion

The difference between the upgaze and downgaze was 10 PD or more (range 10–11 PD) in seven patients (five patients in group B and two patients in group C). Eleven patients had a latent DVD of more than 5 PD (range 6–8 PD; five patients in group A and six patients in group C). Seven patients had SOOA of + 1 (one patient in group A, five patients in group B, and one patient in group C). Forty-five patients had orthophoric eye position with considerably aligned ocular movements postoperatively. The total success rate was 86.5%. Postoperatively, eight of the 10 patients with diplopia experienced a recovery of binocular single vision and three had a recovery of rudimentary stereopsis (Titmus 3000–400 s of arc). Compensatory head posture improved significantly after the surgery. The difference between the degree of the A-pattern, DVD, SOOA, and intorsion in fundus photographs of all groups was significantly pre- and postoperatively (*p* < 0.05) (Table 3). Figure 1 shows a representative case of group A, Fig. 2 shows a case of group B, and Fig. 3 shows a case of group C.

**Table 3:** Pre- and postoperative measurements of the three groups

Parameters	Pre- and post- operation	Eye	Group A: horizontal deviation correction and bilateral superior rectus recession (9)	Group B: horizontal deviation correction with superior oblique muscle lengthening (24)	Group C: horizontal deviation correction with superior oblique muscle lengthening + bilateral superior rectus recession 3 months later (19)
A pattern (PD)	Pre-op		10.2 ± 1.23 (8–12)	32.29 ± 9.3 (16–45)	12.63 ± 2.5 (10–15)
	Post-op		1.10 ± 1.91 (0–5)	7.0 ± 2.43 (3–11)	6.16 ± 2.83 (2–10)
HD (PD)	Pre-op		− 34.0 ± 15.24 (− 15 to − 50)	− 42.91 ± 9.54 (− 20 to − 50)	− 39.47 ± 13.22 (− 15 to − 50)
	Post-op		0.90 ± 3.96 (− 5 to 6)	0.50 ± 4.98 (− 10 to 8)	0.16 ± 3.32 (− 7 to 6)
DVD (PD)	Pre-op	OD	17.40 ± 8.18 (0–30)	7.16 ± 3.86 (0–10)	15.0 ± 11.18 (0–35)
		OS	16.50 ± 9.44 (0–25)	7. 91 ± 2.81 (0–10)	11.95 ± 4.03 (3–20)
	Post-op	OD	3.80 ± 2.10 (0–6)	1.25 ± 1.64 (0–5)	3.58 ± 2.46 (0–8)
		OS	3.80 ± 2.57 (0–7)	2.0 ± 1.76 (0–5)	2.42 ± 2.36 (0–6)
SOOA ( +)	Pre-op	OD	0.90 ± 0.57 (0–2)	2.37 ± 0.76 (1–4)	2.21 ± 0.79 (1–4)
		OS	0.8 ± 0.42 (0–1)	1.20 ± 0.93 (0–3)	1.68 ± 1.11 (0–4)
	Post-op	OD	0.10 ± 0.32 (0–1)	0.16 ± 0.38 (0–1)	0.05 ± 0.23 (0–1)
		OS	0 (0)	0.08 ± 0.28 (0–1)	0 (0)
FI (^°^)	Pre-op	OD	1.40 ± 1.43 (0–4)	13.58 ± 3.80 (6–21)	11.95 ± 4.03 (3–20)
		OS	1.60 ± 1.07 (0–3)	12.3 ± 4.73 (5–20)	10.42 ± 6.0 (2–20)
	Post-op	OD	0.32 ± 0.32 (0–1)	3.28 ± 0.99 (1–5)	2.49 ± 1.20 (1–5)
		OS	0.35 ± 0.24 (0–1)	3.66 ± 1.34 (1–6)	2.37 ± 1.12 (1–5)

Results are shown as mean ± standard deviation (Range)

*PD* prism diopter, *HD* horizontal deviation, *DVD* dissociated vertical deviation, *SOOA* superior oblique overaction, *FI* fundus intorsion, *OD* right eye, *OS* left eye

## Discussion

In the current study, we report the surgical outcomes of different surgical methods based on the extent of patients’ manifestations; the degree of A-pattern, SOOA, and DVD. Taking into consideration, the fundus photography intorsion may support the significance of superior oblique overaction and aid in considering weakening of the superior oblique muscle, for more predictable and better outcomes. The DVD of the patients in group A was more than 15 PD, and the A-pattern was quite small without significant SOOA; hence, the surgical planning was mainly aimed at resolving the DVD by combining superior rectus recession with horizontal alignment, without superior oblique lengthening. However, in group B, the A-pattern was quite large at ≥ 15PD with significant SOOA, but the DVD was ≤ 10PD; the surgical planning was aimed at reducing the A-pattern and SOOA by combining superior oblique lengthening with horizontal alignment. For patients with DVD more than 15PD and significant SOOA that caused obvious intorsion with a large A-pattern, i.e., patients in group C, horizontal correction combined with SO muscle lengthening was performed. In those cases, the DVD was still obvious postoperatively; hence, bilateral superior rectus recession was performed after 3 months or more to correct the residual DVD.

Velez et al. showed that bilateral superior rectus muscle recession can correct small amounts of A-pattern; larger amounts of A-pattern required additional SO weakening [3]. Ganesh et al. reported that small to moderate A patterns measuring less than 20PD may respond well to superior rectus weakening with SO weakening [4]. Ha et al. suggested that horizontal muscle surgery alone should be performed when the degree of SOOA and the A-pattern deviation are minimal [5]. In terms of the A-pattern, our findings were in line with those of prior studies.

For the management of DVD, superior rectus recession is usually the preferred surgical method [10], while weakening of the SO muscle (tendon lengthening, tenotomy, or tenectomy) is found to be effective in collapsing of the A-pattern [2]. Previous studies have shown good results in correcting the DVD with the A-pattern and SOOA by superior rectus recession with posterior tenectomy of the superior oblique [2–4]. In cases of pattern deviations with oblique muscle overaction, oblique muscle weakening may be a suitable option as it decreases the torsion that may contribute to the pattern [11]. A large reduction in intorsion was reported by Wu et al. after the weakening of the SO muscle [12]. In our series, 5 out of 9 patients in group A had a little degree (10–11PD) of DVD after surgery. The incomitance of DVD across horizontal gazes was not taken into account in our investigation. According to McCall et al., superior rectus weakening alone will not be able to resolve the incomitance exhibited when DVD is present, and undercorrection may result [13]. It's possible that, in addition to superior rectus recession, adding superior oblique weakening when DVD is greatest in abduction or inferior oblique anterior transposition when DVD is greatest in adduction would have yielded better results for such patients [14].

The A-pattern may be associated with SOOA and intorsion, but ocular torsion may not be the primary etiological factor for pattern strabismus [15]. However, whenever there is obvious hyperfunction of the SO muscle and fundus intorsion, the SO muscle should be weakened [16]. This can be achieved by lengthening the SO muscle by non-absorbable sutures, which can partially weaken the function of the SO muscle in different grades [17]. Partial resection of the SO tendon is sometimes used to deal with the rotation effect (anterior part of the tendon) or the vertical effect (posterior part of the tendon) alone. However, in the case of the Helveston triad, the vertical effect (to correct the DVD) and the rotation effect (to correct the A-pattern) should be managed, thereby requiring tenectomy. Moreover, the reduction in the A-pattern obtained after partial resection of the posterior fibers of the SO muscle is less than that obtained by complete resection (tenotomy or tenectomy) [18]. Certain substances have been used to separate the broken ends of the SO muscle, such as Achilles tendon [19] or a piece of silicon tape [20], but adding these substances would increase the time and complexity of the surgery. The silicone expanders may cause a severe inflammatory reaction [21]. A direct method is to use a 3–8 mm polyester suture between the broken ends according to the results of the objective forced duction test during the surgery.

Good correlations were found between fundus intorsion, SOOA, and A patterns [22]. Preexisting intorsion in five of eight cases of A-pattern was observed by Sharma et al. These five cases also had SOOA, and the other three cases did not have SOOA and did not show intorsion. This indicates a direct cause–effect relationship between torsion and SOOA [23]. Fundus torsion is described as a marker of SOOA. In the present study, patients with SOOA showed varied degrees of fundus intorsion, particularly when SOOA was obvious. We believe that this should be taken into consideration when planning the surgery.

Among previous studies investigating Helveston syndrome, Ha et al. reported a success rate of 57.1% [5], whereas Wu et al. reported a success rate of 53.3%, with a small amount of eso-drift in the primary position postoperatively [6]. The overall success rate in the present study was 86.5%, which is higher than those of previous studies; no SO palsy, postoperative inverted V pattern, or induced extorsion were noted.

Nevertheless, there are some limitations in this study. First, the study was retrospective in nature. Second, the follow-up period was relatively short, and the sample size was small. Third, there was no control group, and the surgical techniques employed in the study were variable. A prospective randomized controlled clinical trial is recommended. Patients in group C were managed in two different sessions. Although staging surgery for strabismus involving horizontal and vertical eye movements has been recommended, it may be better if all three surgical methods (lateral rectus recession, superior rectus recession, and superior oblique muscle lengthening) were performed at the same time, as this would reduce the psychological and financial burden on patients.

In conclusion, our experience with the above-mentioned case classification revealed that with such a peculiar combination of ocular motility disturbances, satisfactory outcomes can be achieved in terms of DVD reduction in the primary position, as well as the collapse of the A-pattern and SOOA. The surgical planning of Helveston syndrome should be designed based on the degree of the A-pattern, SOOA, DVD, and the intorsion in fundus photographs, and the appropriate approach should be selected to improve patient satisfaction.

## Data Availability

The datasets generated and/or analyzed during the present study are available from the corresponding author upon reasonable request.
